# State of the art in parallel ankle rehabilitation robot: a systematic review

**DOI:** 10.1186/s12984-021-00845-z

**Published:** 2021-03-20

**Authors:** Mingjie Dong, Yu Zhou, Jianfeng Li, Xi Rong, Wenpei Fan, Xiaodong Zhou, Yuan Kong

**Affiliations:** 1 Beijing Key Laboratory of Advanced Manufacturing Technology, Faculty of Materials and Manufacturing, Beijing University of Technology, No.100, Pingleyuan, Chaoyang District, Beijing, 100124 China; 2 Department of Neurology, the Affiliated Hospital of Qingdao University, 59 Haier Road, Laoshan District, Qingdao, 266000 China; 3 Beijing Institute of Control Engineering, Beijing, 100094 China

**Keywords:** Parallel ankle rehabilitation robot, Mechanism configurations, Trajectory tracking control, Rehabilitation training

## Abstract

**Background:**

The ankle joint complex (AJC) is of fundamental importance for balance, support, and propulsion. However, it is particularly susceptible to musculoskeletal and neurological injuries, especially neurological injuries such as drop foot following stroke. An important factor in ankle dysfunction is damage to the central nervous system (CNS). Correspondingly, the fundamental goal of rehabilitation training is to stimulate the reorganization and compensation of the CNS, and to promote the recovery of the motor system’s motor perception function. Therefore, an increasing number of ankle rehabilitation robots have been developed to provide long-term accurate and uniform rehabilitation training of the AJC, among which the parallel ankle rehabilitation robot (PARR) is the most studied. The aim of this study is to provide a systematic review of the state of the art in PARR technology, with consideration of the mechanism configurations, actuator types with different trajectory tracking control techniques, and rehabilitation training methods, thus facilitating the development of new and improved PARRs as a next step towards obtaining clinical proof of their rehabilitation benefits.

**Methods:**

A literature search was conducted on PubMed, Scopus, IEEE Xplore, and Web of Science for articles related to the design and improvement of PARRs for ankle rehabilitation from each site’s respective inception from January 1999 to September 2020 using the keywords “ parallel”, “ ankle”, and “ robot”. Appropriate syntax using Boolean operators and wildcard symbols was utilized for each database to include a wider range of articles that may have used alternate spellings or synonyms, and the references listed in relevant publications were further screened according to the inclusion criteria and exclusion criteria.

**Results and discussion:**

Ultimately, 65 articles representing 16 unique PARRs were selected for review, all of which have developed the prototypes with experiments designed to verify their usability and feasibility. From the comparison among these PARRs, we found that there are three main considerations for the mechanical design and mechanism optimization of PARRs, the choice of two actuator types including pneumatic and electrically driven control, the covering of the AJC’s motion space, and the optimization of the kinematic design, actuation design and structural design. The trajectory tracking accuracy and interactive control performance also need to be guaranteed to improve the effect of rehabilitation training and stimulate a patient’s active participation. In addition, the parameters of the reviewed 16 PARRs are summarized in detail with their differences compared by using figures and tables in the order they appeared, showing their differences in the two main actuator types, four exercise modes, fifteen control strategies, etc., which revealed the future research trends related to the improvement of the PARRs.

**Conclusion:**

The selected studies showed the rapid development of PARRs in terms of their mechanical designs, control strategies, and rehabilitation training methods over the last two decades. However, the existing PARRs all have their own pros and cons, and few of the developed devices have been subjected to clinical trials. Designing a PARR with three degrees of freedom (DOFs) and whereby the mechanism’s rotation center coincides with the AJC rotation center is of vital importance in the mechanism design and optimization of PARRs. In addition, the design of actuators combining the advantages of the pneumatic-driven and electrically driven ones, as well as some new other actuators, will be a research hotspot for the development of PARRs. For the control strategy, compliance control with variable parameters should be further studied, with sEMG signal included to improve the real-time performance. Multimode rehabilitation training methods with multimodal motion intention recognition, real-time online detection and evaluation system should also be further developed to meet the needs of different ankle disability and rehabilitation stages. In addition, the clinical trials are in urgent need to help the PARRs be implementable as an intervention in clinical practice.

## Background

**Fig. 1:**
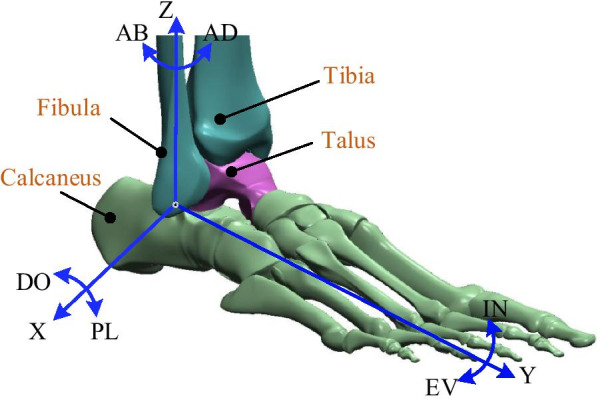
Anatomy of AJC and its rotational motions [1]

The ankle joint complex (AJC) mainly consists of the tibia, fibula, talus and calcaneus, as shown in Fig. 1 [1]. The tibia and fibula are considered one unit to simplify the motions of the AJC, and the ankle joint involves the articulation between the tibia-fibula unit and the talus [1]. The AJC is of fundamental importance for balance, support, and propulsion. However, it is particularly susceptible to musculoskeletal and neurological injuries, especially neurological injuries such as the drop foot following stroke. Based on a report from the American Heart Association, approximately 795,000 people experience stroke in the United States each year [2]; stroke has poor prognosis and is associated with a high proportion of patients with drop foot, becoming the leading cause of permanent disabilities worldwide, with over 15 million new cases each year and 50 million stroke survivors [3]. An important factor in ankle dysfunction is damage to the central nervous system (CNS), which needs to be stimulated for reorganization and compensation and to promote the recovery of the motor system’s motor perception function [4]. Therefore, physiotherapy becomes essential for patients under this circumstance [5, 6]. During treatment, patients can regain their limited range of motion (ROM), restrengthen weak muscles, recover dynamic balance, and thus gradually restore motion functions [7]. However, this necessitates a long, repetitive, and intensive rehabilitation process, leading to a large burden and workload on the traditional ankle rehabilitation training, which is performed by therapists on a one-on-one hands-on basis to gradually stimulate and repair the damaged CNS [8]. In addition, traditional ankle rehabilitation training cannot provide sufficient training frequency and intensity due to limited time and resources [6]. Moreover, the rehabilitation training plans are developed based on therapists’ subjective clinical experience, which leads to the problem where therapists cannot accurately control the changes in complex forces, rehabilitation training forms and training parameters; hence, it is difficult to ensure accurate training of the affected limbs.

Therefore, it is of vital importance to replace traditional ankle rehabilitation training by developing ankle rehabilitation robots, which have the benefits of providing long-term accurate and uniform rehabilitation training as well as the ability to adaptively modify the difficulty of rehabilitation training according to real-time feedback from training [9]. In addition, the application of robot-assisted ankle rehabilitation techniques allows real-time data collection throughout the training process to further determine the accuracy of the training [10], therein allowing an assessment of the biomechanical properties of the AJC [11] and mobility [12] to customize future treatments [13]. Particularly, these techniques can effectively reduce the labor intensity of medical staff, improve the effect of ankle rehabilitation training and compensate for the shortage of rehabilitation medical resources. The parallel ankle rehabilitation robots (PARRs) and wearable devices are the most studied technologies for ankle rehabilitation, in which, the PARRs have a fixed platform and can be used for multiple degree of freedom (DOF) rehabilitation with a small size and high rigidity, while wearable devices are known as exoskeleton or powered orthoses and are often used for gait training. The advantage of a PARR compared with a wearable device is that the lower leg will not follow the swing during the process of ankle rehabilitation training, which can allow avoiding a secondary injury to the ankle joint.

Various reviews of robotic devices for ankle rehabilitation have been performed [6, 14–17]. However, references [6, 15–17] mainly focused on the mechanical design of ankle rehabilitation robots and covered both PARRs and wearable robots, while reference [14] focused on the effectiveness of robot-assisted therapy on ankle rehabilitation with both PARRs and wearable ones, and they concluded that wearable robots are more suitable for gait training, while PARRs are better suited for ankle exercises. Therefore, there is no systematic and comprehensive review specifically on the development of PARRs. With increasing research on PARRs, different mechanism configurations, actuator types, and rehabilitation training methods have been proposed, which may present a challenge to researchers new to this field.

The purpose of this paper is to provide a systematic review of the state of the art in PARR technology, with consideration of the mechanism configurations, actuator types, and rehabilitation training methods, thus serving as a tutorial for engineers who will design or control PARRs, making them aware of the advantages and limitations of different mechanical and control choices. From a research point of view, this paper also proposes a taxonomy expansion and a review of future research directions. The following sections review the PARRs with regard to the mechanism configurations, actuator types with different trajectory tracking control techniques, and rehabilitation training methods; we also compare, analyze and summarize them separately. Finally, the research hotspots and trends are discussed, and we present the takeaways regarding PARRs as the findings of this review.

## Methods

### Search strategy

The systematic review was conducted by performing a literature search with PubMed, Scopus, IEEE Xplore, and Web of Science, and the search was limited to English-language articles (i.e., journal articles and conference proceedings) published from January 1999 to September 2020. The reason that we chose January 1999 as the starting point for searches is that the first PARR was developed in 1999, named “ Rutgers Ankle” [18]. The electronic search keywords were “ parallel”, “ ankle” and “ robot”. Appropriate syntax using Boolean operators and wildcard symbols was used for each database to include a wider range of articles that may have used alternate spellings or synonyms.

### Inclusion criteria

Studies were eligible for inclusion if the following criteria were met:

- The device described is a parallel mechanism with at least 2-DOFs;
- The study focuses on ankle rehabilitation;
- The PARRs involved are developed with prototypes, not simply theoretical concept designs;
- The involved paper is a scientific article written in the English language and accessible to the authors.

### Exclusion criteria

- Devices such as wearable ankle rehabilitation robots, ankle-foot prostheses and 1-DOF robotic ankle rehabilitation systems were excluded;
- Studies not related to ankle recovery were excluded as well;
- Studies with insufficient information on the PARR design were excluded;
- Non-English articles were excluded.

### Data extraction

**Fig. 2:**
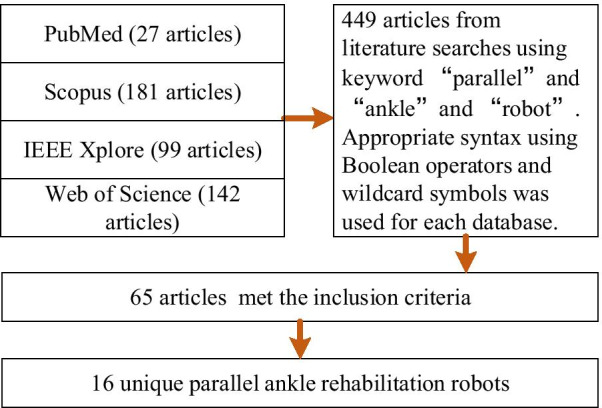
Flow diagram of the literature search and results

The final search queries for the review were completed on September 30, 2020. A total of 65 articles representing 16 unique PARRs met the inclusion criteria, and the associated full articles were obtained by downloading them from electronic databases. Inside the selected articles, 57 out of the 65 ($87.7\%$) articles were published after 2009, which, combined with the increasing rate of stroke survivors, shows that research on PARRs has been very hot in the last decade. A summary of the article selection procedure can be seen in Fig. 2. During the article selection, we learned that many PARRs can realize ankle rehabilitation training in some DOFs through different mechanism configurations. In addition, only two actuator types have been found for actuating PARRs, i.e., pneumatic and electrically driven, which make full use of their respective advantages. For smooth and precise PARR control, many trajectory tracking control methods have been utilized to improve the control system. On this basis, different rehabilitation training methods have been realized and combined with some advanced control algorithms to improve the safety and comfort. Next, the selected articles with different PARRs are summarized in terms of the mechanism configurations, actuator types with different trajectory tracking control techniques, and rehabilitation training methods to ease their comparison.

## Results

**Fig. 3:**
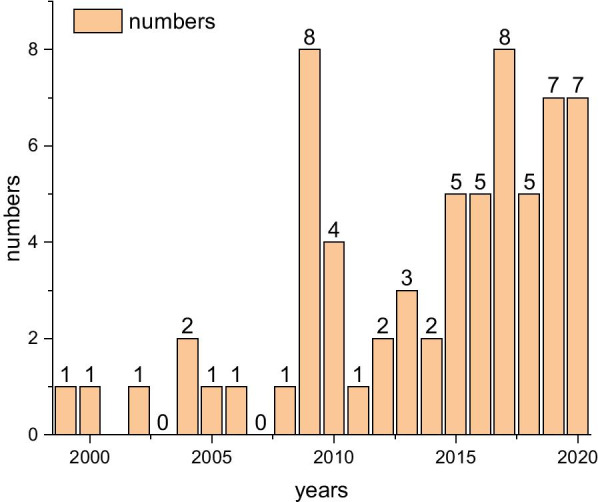
Number of the selected publications on PARRs from January 1999 to September 2020

As shown in Fig. 1, the AJC is capable of moving in 3-DOFs, namely, dorsiflexion/plantarflexion (DO/PL), inversion/eversion (IN/EV), and adduction/abduction (AD/AB). The data of the typical motion limits along these directions, as determined in an in vitro study by Siegler et al., are reproduced, as in [19]. Therefore, the mechanical design of the PARR should match the 3-DOFs of the AJC and satisfy all ROMs as much as possible. The searching of various scientific databases reveals an increase in the number of peer-reviewed publications on PARRs, as shown in Fig. 3.

### Mechanism configurations

**Fig. 4:**
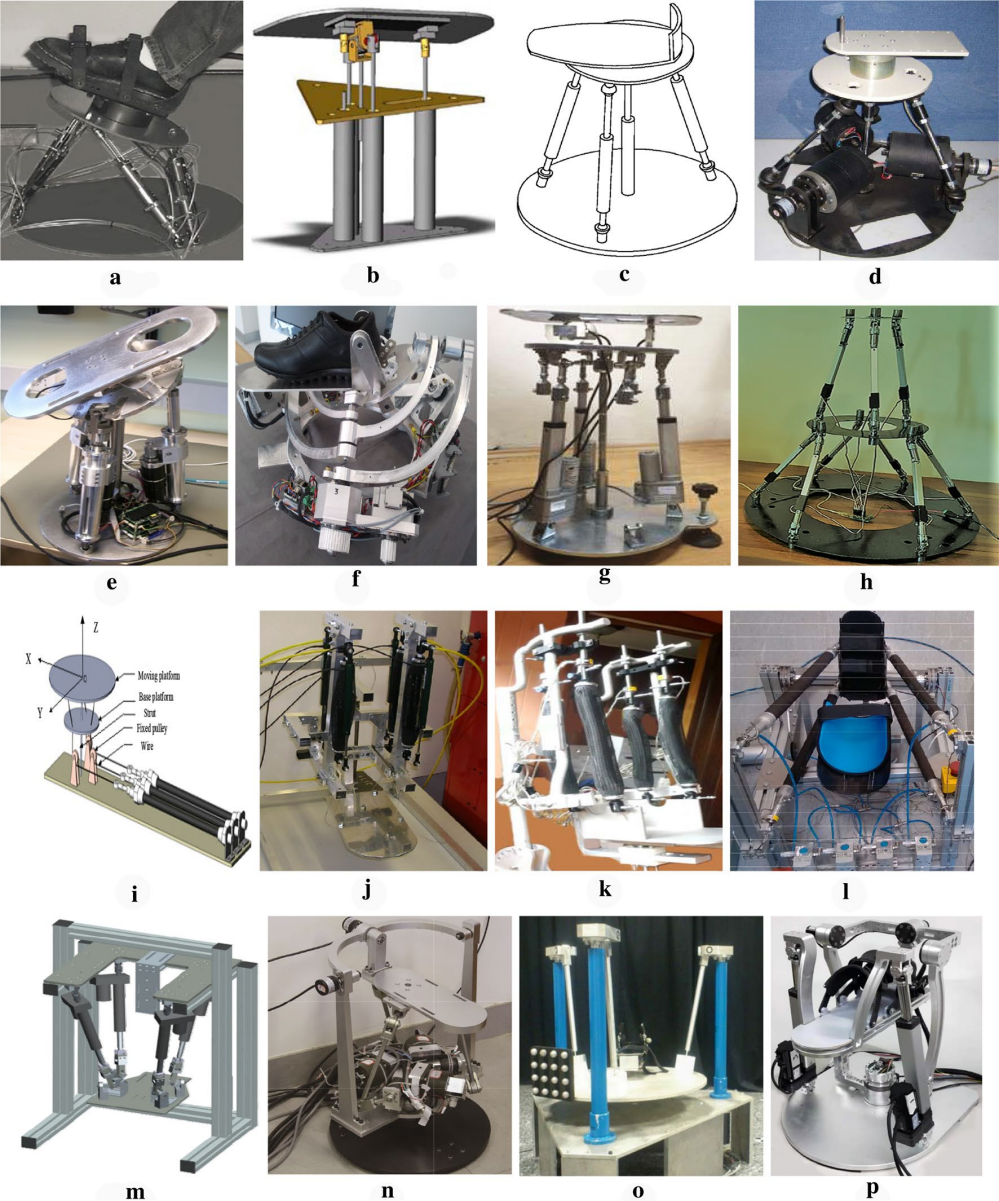
PARRs with different mechanism configurations. **a** Rutgers Ankle -“ Rutgers University”; **b** Reconfigurable ankle robot -“ Gwangju Institute of Science and Technology”; **c** 3- SPS/SP mechanism -“ King’s College London”; **d** 3- RSS/S mechanism -“ Heibei University of Technology”; **e** ARBOT -“ IIT”; **f** PKAnkle -“ Institute of Industrial Technologies and Automation”; **g** 2-DOFs parallel ankle robot -“ Karadeniz Technical University”; **h** 9-DOFs hybrid PARR -“ University of Birmingham”; **i** 2-DOFs ankle rehabilitation robot -“ Wuhan University of Technology”; **j** Soft parallel robot(SPR) -“ University of Auckland”; **k** Compliant ankle robot -“ Nazarbayev University”; **l** Compliant ankle rehabilitation robot -“ University of Auckland”; **m** Redundantly actuated ankle robot -“ University of Auckland”; **n** 3-RUS/RRR -“ Beijing Jiaotong University”; **o** 3- PRS -“ Universidad Politécnica de Valencia”; **p** 2-UPS/RRR ankle rehabilitation robot -“ Beijing University of Technology”

To satisfy the workspace of the AJC for rehabilitation, different mechanism configurations of PARRs have been developed, e.g., the Rutgers Ankle, which was based on the Stewart platform, as shown in Fig. 4a [18], and its derivatives [20]; the 4-DOF reconfigurable PARR shown in Fig. 4b, which can generate the pitch motion of each platform, the roll and heave motions ($1T-3R$) or pitch motion of each platform, and two translational motions ($2T-2R$) at both platforms [21], where *T* represents translation while *R* represents rotation [22]; the $3{\mathrm{- }}SPS/SP$ parallel mechanism shown in Fig. 4c, where *S* and *P* represent spherical and prismatic joints, respectively [23]; the 3-DOF $3{\mathrm{- }}RSS/S$ parallel mechanism shown in Fig. 4d, where *R* stands for the revolute joint [24]; the 2-DOF 3UPS/U overactuated parallel mechanism named ARBOT shown in Fig. 4e [25, 26], where *U* stands for the universal joint, and an underlined letter here represents the actuated joint [27, 28]; the adaption of Gosselin’s spherical robot, named PKAnkle, shown in Fig. 4f [86]; and some other parallel mechanisms developed for ankle rehabilitation, as shown in Fig. 4g [55], Fig. 4h [29, 30], and Fig. 4i [52].

The abovementioned PARRs all have the advantages of parallel mechanisms, including low inertia, high rigidity, compactness, greater portability, and precise resolution compared with serial robots. They also have another thing in common, i.e., their actuators are below their footplate, which means that the mechanism axes of rotation are far offset from the AJC axes of rotation although this makes the structure and actuation much simpler, readily causing unexpected movements for patients, such as translations induced by rotations, which is much worse for a patient whose shank cannot move arbitrarily. This can cause the AJC to be subjected to uneven random forces during rehabilitation, vulnerable to secondary damage. By contrast, a patient can keep his/her ankle stationary and fully relaxed on devices actuated from above and can more easily make the rotation center of the PARR coincide with that of the AJC, which can avoid the secondary injury of AJC to a certain extent.

For this reason, many PARRs of these types have been developed with both pneumatic-driven and electrically driven actuation, such as the 3-DOF pneumatic-driven soft parallel robot (SPR) shown in Fig. 4j [31]; the 3-DOF pneumatic-driven intrinsically compliant PARR shown in Fig. 4k [32, 33]; the pneumatic-driven compliant ankle rehabilitation robot (CARR) shown in Fig. 4l [34]; the 3-DOF redundant electrically driven PARR shown in Fig. 4m [35, 36]; the 3-DOF 3-RUS/RRR redundantly actuated parallel mechanism shown in Fig. 4n [37, 38], which can also be changed into a 3-RUS/U redundant mechanism with 2-DOFs when the redundant actuator of the constraint limb is self-locked [39]; the 3-DOF 3-PRS parallel manipulator for ankle rehabilitation shown in Fig. 4o [40–42]; and the 3-DOF 2-UPS/RRR PARR shown in Fig. 4p [43–46].

For all these PARRs, the most commonly used actuator of pneumatic-driven PARRs is the pneumatic muscle actuator (PMA) due to its superiority in terms of low weight and high power/volume ratios, which can provide an intrinsic softness to make joint compliance possible [47, 70]. However, the PMA can only pull and cannot push, which leads to a complexity of the mechanism in that $(n+1)$ actuators are required to achieve *n*-DOF motion of the PARR. By contrast, the electric motor can be easily controlled to rotate in the forward and reverse directions accurately, which means that there is no need for the electrically driven PARRs to be driven redundantly; in addition, they can have a smaller size while achieving the same function as that of the pneumatic-driven PARRs. However, the compliance of an electrically driven PARR needs to be realized by adopting compliance control algorithms [27, 60–62]. In addition, many PARRs adopt actuation redundancy to eliminate singularities and to improve the dexterity for isotropic force distribution, such as the ARBOT [28] and the 3-RUS/RRR PARR [37, 38]. However, this also leads to the complex mechanism configuration that is difficult to control, and a branch chain that is easy to interfere with.

### Actuator types with different trajectory tracking control techniques

As we summarized above, there are two types of PARRs based on actuator type, namely, pneumatic-driven PARRs and electrically driven PARRs. The PARRs of Fig. 4a–l are all pneumatic-driven PARRs and driven by PMAs due to their superior features in terms of their low weight, high power/volume ratios and intrinsic softness, which enable joint compliance [47]. The remaining PARRs are all electrically driven ones. To help a patient regain his/her limited ROM, the PARR needs to drive the AJC to be trained along a predetermined trajectory, which means that the kinematic chains of the robot should provide excellent trajectory tracking control performance, especially for the pneumatic-driven PARRs. To analyze the nonlinear and time-varying characteristics of PMAs, Jamwal et al. developed an optimal fuzzy dynamic model of the pneumatic muscle to accurately predict muscle behavior [48]. On this basis, a fuzzy-based disturbance observer (FBDO) was proposed to address the nonlinear characteristics of the PMA, and an adaptive fuzzy logic controller based on the Mamdani inference was developed and appended with the FBDO to compensate for the transient nature of the PMA, achieving very good trajectory tracking performance [49]. In addition, the optimal path of a PARR was calculated by minimizing the joint reaction moments and the tension along ligaments and muscle-tendon units, to help generate more reasonable rehabilitation training trajectories [50]. Using the CARR as the platform, Meng et al. proposed a robust normalized iterative feedback tuning (NIFT) technique for its repetitive training control and proposed a multi-DOF normalized IFT technique to increase the controller robustness by obtaining an optimal value for the weighting factor and offering a method with learning capacity to determine optimal controller parameters [51]. Similarly, Ai et al. developed an adaptive backstepping sliding mode control (ABS-SMC) method to solve the nonlinear characteristics of the pneumatic muscles during operation and to address the human-robot uncertainties in rehabilitation, with five healthy subjects involved to verify the robustness of the controller; the maximum error, average error and root mean square deviation (RMSD) were $7.05{\mathrm{\% }}$, $2.15{\mathrm{\% }}$ and $0.78^{\circ }$, respectively [52]. The research group also developed a multi-input-multi-output disturbance compensated sliding mode controller (MIMO-DCSMC) to tackle the unmodeled uncertainties and the coupling interference that existed in the synchronous movement of multiple PMs [53]. In addition, Zhang et al. developed a cascade controller to improve the trajectory tracking accuracy of their CARR, with position control in the outer loop and pneumatic muscle force control in the inner loop; here, the normalized root mean square deviation (NRMSD) of the trajectory tracking could be less than 2.3% [54].

Through the abovementioned summary, we can see that most pneumatic-driven PARRs were developed with trajectory tracking control to improve rehabilitation accuracy [48, 49, 51, 52, 54] because the PMAs are highly nonlinear and require accurate modeling to be precisely controlled [54]. However, the precise control of PMAs is a challenging problem due to their nonlinear and time-varying characteristics. Specifically, they can be modeled as a parallel connection of a nonlinear friction force, a nonlinear spring, and a nonlinear contractile element. It is difficult to identify the coefficients associated with these elements with precision, as they change along the course of continuous use [73]. Therefore, trajectory tracking control is of vital importance for pneumatic-driven PARRs. To address the nonlinear characteristics of PMAs, the FBDO, NIFT, ABS-SMC and adaptive trajectory tracking control strategies were developed in [49, 51, 52, 54]. By contrast, trajectory tracking control is not required for electrically driven PARRs under normal circumstances because all kinematic branches are controlled by the servo motor, and the electrically driven PARRs can achieve high-precision trajectory tracking control based on the position controller [24, 28, 35, 60]. The only trajectory tracking algorithm used on an electrically driven PARR is given in [55]; it uses a fractional order PID (FOPID) and a plug-in-type repetitive controller cascaded to a PID controller [56] to resist external disturbances during human-robot interactions and reduce the side effects during ankle rehabilitation training because the motors are not servo motors with closed-loop control.

### Rehabilitation training methods

The main advantage of automated rehabilitation systems is the ability to perform a large number of repetitions, which has been proven to be extremely beneficial in the treatment of neuromuscular injuries [57]. Therefore, rehabilitation training methods based on different control strategies are of vital importance and have been developed on different PARRs. To provide control algorithms for most of the exercises suggested by standard rehabilitation protocols, including passive and active training with effective assistive and resistive capabilities, Saglia et al. designed a control framework for ARBOT, as shown in Table 1 [28, 60], which is the most commonly used method for ankle rehabilitation. Ankle rehabilitation mainly includes the training of the ROM, muscle strength and proprioceptive sensing. Of these, the ROM training is the basic function of all PARRs, and is usually conducted through trajectory tracking control techniques, during which the AJC is driven along a predefined trajectory circularly in the early stage of therapy, thus enabling patients to regain their limited ROM. Ankle muscle strength training is achieved through active rehabilitation, such as isometric exercise, isotonic exercise or isokinetic exercise, usually when the patient’s AJC already has some muscle strength. The last stage of the ankle rehabilitation process is proprioceptive training, at which point the patient has almost fully regained his/her ROM with a certain amount of muscle strength. Balance exercises are typical for this kind of training, and in this case, the patient has to stand on top of the ankle rehabilitation robot and try to keep his/her balance, as if he/she is using a wobble board.

**Table 1:** Control framework for rehabilitation exercises [28, 60]

Class of exercise	Exercise mode (patient)	Control algorithms
ROM	Passive	Position control
	Active	Assistive control
Strength training	Active (isometric)	Position control
	Active (isotonic)	Admittance control
Proprioceptive training	Active	Hybrid control

For the electrically driven PARRs, Yoon et al. proposed a wide set of exercise modes to improve rehabilitation training, ranging from passive exercise to proprioceptive training such as balance exercise based on position and impedance control theories [59]. The ARBOT is designed to be actuated by custom-designed servo linear actuators, with a position control scheme to realize patient-passive exercise and admittance control to realize patient-active exercise. However, it does not take the compliance control of passive exercise into consideration; only assistive control has been experimentally evaluated, with other control strategies only being analyzed and simulated [27, 60]. For the 3-DOF redundantly actuated PARR, to ensure system safety and effective rehabilitation, Tsoi et al. developed a variable-impedance controller using computer simulations considering the computer models for both the robot and the human AJC, with the impedance parameters selected by referring to the ankle compliance as determined by the ankle model under corresponding foot configurations, therein showing that varying the manipulator impedance in proportion to the ankle compliance does indeed provide a performance advantage over constant impedance control [61, 62]. On this basis, a force-based impedance controller that considers the coupling between various DOFs of a PARR was proposed in [63], which utilizes coupling information from the manipulator Jacobian and inertia matrix and therefore does not require precise dynamic modeling and measurement of the end effector velocity, showing good performance in trading off the positional accuracy to maintain the safety of the PARR [64]. However, the robot suffers from a problem whereby unexpected loads may be exerted on a patient’s foot when rehabilitation begins [37]. For the 3-DOF 2-UPS/RRR PARR, the passive rehabilitation training trajectories were preset with the control system built [65, 66], and three compliance rehabilitation training strategies developed based on admittance control and its derivatives, namely, patient-passive compliance exercise, isotonic exercise, and patient-active exercise, were developed to enhance the training safety, fully considering the patient’s muscle strength level and covering different stages of recovery with good compliance [67].

For the pneumatic-driven PARR, the Rutgers Ankle adopted a position controller for passive training to drive the patient’s AJC along certain trajectories and a force controller for active resistive exercise [18, 58], for which four subjects participated in proof-of-concept trials [58]. In addition, many researchers have studied compliance control strategies and different rehabilitation training modes, although their actuators have intrinsic softness to achieve joint compliance. Jamwal et al. developed an interactive training paradigm based on impedance control for their intrinsically compliant PARR, which allows patients to modify robot-imposed motions according to their own level of disability, based on which four training modes with different impedance were developed [68]. To overcome the dependence of the designed impedance control scheme on the decisions of physical therapists, an adaptive impedance control scheme for adapting the PARR impedance according to patients’ active participation was further proposed to provide customized robotic assistance [69]. Liu et al. studied the compliance control of the CARR, including a low-level compliance adjustment controller in joint space and a high-level admittance controller in task space [70]. To better improve the effectiveness and safety of the CARR, Zhang et al. further studied the adaptive patient-cooperative control strategy based on a variable-parameter admittance controller, in which an admittance controller was used to adaptively modify the predefined trajectory according to real-time ankle measurements; and the passive mode based on a joint-space position controller, patient-robot cooperative mode based on a fixed-parameter admittance controller, and cooperative mode based on a variable-parameter admittance controller were all examined [71]. Similarly, Ayas et al. developed a fuzzy-logic-based adaptive admittance control scheme for their PARR to adapt the resistance/assistance level to the patient’s disability level, with both active ROM exercises and isotonic exercises [72].

Through the abovementioned summary, we can see that safety is always the most important for robot-assisted neurological rehabilitation, not only compliance control in the task space but also compliance of the actuators and the robot itself are of crucial importance to ensure safety, because the patients can provide moderate torque to initiate the active motion when they have certain muscle strength, and excessive interaction forces may occur in this situation if the PARR is not flexible. In this respect, pneumatic-driven PARRs have more advantages because the pneumatic muscle has a lightweight and compliant nature, which makes it more appropriate for natural interaction with patients [74]. On this basis, many pneumatic-driven PARRs have been developed based on further studies on compliance control, such as impedance control [68], adaptive impedance control [69], admittance control [70], and adaptive admittance control [71], which can adaptively modify the predefined trajectory based on real-time measurements of the human-machine interaction force. In contrast, compliance control is a very important research hotspot for electrically driven PARRs because their actuators do not have intrinsic compliance features. Many compliance control algorithms have been studied for this type of PARR, such as admittance control [27, 60] and adaptive admittance control [72]. Impedance control, admittance control and their extensions will always be key points of research for years to come for the human-robot interaction in all rehabilitation robot fields.

## Discussion

### Similarities among the reviewed PARRs

#### Mechanical design and mechanism optimization

The aim of PARRs is to improve ankle rehabilitation performance, therein assisting the AJC in regaining its ROM and muscle strength. Therefore, a designed PARR must have a sufficient workspace and excellent kinematic performance, such as velocity transfer performance and force transfer performance. Because the AJC has 3-DOFs, many PARRs are designed with 3-DOFs, such as the PARRs in [24, 32, 34, 35, 37, 40, 43–45], to satisfy all the motion of the AJC. Some PARRs are designed to have only 2-DOFs to simplify the mechanism by eliminating the AD/AB motion, such as the PARRs in [25, 55]. In addition, if the links between the two platforms of the PARRs are parallel to each other (i.e., if the connection points on both platforms are symmetrical), the PARRs can result in a singular configuration, and the design of the PARRs is complex and calls for obtaining a tradeoff between several conflicting objectives such as the minimization of actuator forces versus the maximization of the workspace while maintaining a close-to-unity condition number, etc. Therefore, both the pneumatic-driven and electrically driven PARRs utilize a kinematic design and optimization in the configuration of the parallel mechanisms. Jamwal et al. utilized many algorithms to optimize the kinematic design, actuation design and structural design of their PARRs, such as the modified genetic algorithms [31], evolutionary-algorithm-based nondominated sorting algorithm [75], modified differential evolution algorithms [38], biased fuzzy sorting genetic algorithm (BFSGA) [32], and fuzzy-dominated sorting evolutionary algorithm approaches [33], to provide a better discrimination among solutions and thereby improve the design of the PARRs. They also utilized a modified fuzzy inference system (FIS) to solve the forward kinematics (FK) problem, as parallel robots exhibit highly coupled nonlinear motions; hence, a unique closed-form solution of their FK cannot be obtained [36]. Wang et al. proposed a modified differential evolution (DE) algorithm to solve the multiobjective optimization problem, including the occupied space, input/output transmission and torque performance, and multicriteria constraints [38]. We can also see from the above review results that there are two types of PARRs based on the mechanism configurations: those with the actuator from below the PARRs and those with the actuator above the PARRs. Although the actuator from below the PARRs makes the structure and actuation much simpler, the mechanism axes of rotation are far offset from the AJC axes of rotation, hard to make the rotation center of the PARR coincide with that of the AJC, which can cause the AJC to be subjected to uneven random forces during rehabilitation, vulnerable to secondary damage. By contrast, a patient can keep his/her ankle stationary and fully relaxed on devices actuated from above and can more easily make the rotation center of the PARR coincide with that of the AJC, which can avoid the secondary injury of AJC to a certain extent, and this has been the trend in newly designed PARRs.

In conclusion, there are three main considerations for the mechanical design and mechanism optimization of PARRs. The first is to choose a better actuator type, whether pneumatic-driven or electrically or whether the actuator is below or above the PARRs. The second is to cover the all 3-DOFs of the AJC, thus ensuring that one PARR can effectively carry out rehabilitation training in the DO/PL, IN/EV and AD/AB DOFs. The third is to optimize the kinematic design, actuation design and structural design of the PARRs to make them more rational to use in rehabilitation training.

#### Trajectory tracking and interactive control

From the results of trajectory tracking control techniques and the rehabilitation training methods, we can see that all of these are related to the ankle rehabilitation training modes. In this regard, the control framework of Saglia et al., as shown in Table 1, is the most systematic [28, 60]. Trajectory tracking control is used to help the PARR drive the AJC along the obtained trajectory. On the one hand, it is used in passive rehabilitation training, during which the AJC is driven along a predefined trajectory circularly to help patients regain their limited ROM, and trajectory parameters such as the wave type, speed, amplitude and number of repetitions can be set by physicians according to the patient’s degree of recovery, as in [52, 54–56]. On the other hand, trajectory tracking control is mainly used in the inner-position loop control of the admittance control to track the deduced angle output of the PARR based on the measured interactive force/torque and the admittance control algorithm, as in [60, 67, 71]. The interactive control is mainly used in active rehabilitation training, which involves human participation and is often based on force/torque sensors to help a patient further restore his/her AJC ROM, muscle strength and proprioceptive sense. For this interactive control, impedance control [61, 68, 69] and admittance control [60, 67, 71, 72] are the most commonly used, which can improve the compliance of the control system, improve the enthusiasm of patients to participate in ankle rehabilitation training, and improve the effect of rehabilitation training.

In addition to these, joint force control [63] and hierarchical compliance control [70] have also been used on PARRs to improve passive rehabilitation training and active rehabilitation training. However, all the control strategies and rehabilitation training modes are in the stage of testing, with none being used in clinical applications.

In brief, the trajectory tracking and interactive control are both used for the different rehabilitation training methods. On the one hand, the trajectory tracking accuracy must be high enough to drive the AJC along the required trajectory precisely, which may be somewhat difficult for the pneumatic-driven PARRs due to the nonlinearity of the PMAs and is the reason why many trajectory tracking control algorithms, such as the FBDO, NIFT, and ABS-SMC [49, 51, 52, 54], have been developed. On the other hand, interactive control algorithms such as impedance control, admittance control and their derivatives are used for active rehabilitation to effectively stimulate a patient’s active participation and ensure training safety [60, 61, 67–69, 71, 72].

### Differences among the reviewed PARRs

Based on the abovementioned systematic review and summary, we can see that many core aspects of PARRs have converged on common solutions, especially for PARRs using the same actuator types, even though their mechanical designs are often quite different. The parameters of the 16 abovementioned PARRs are summarized in detail with their differences in Table 2. Particularly, we have made the order of the PARRs in Table 2 exactly the same as that in Fig. 4, which, combined together, can better show the differences and comparisons among the reviewed PARRs. In addition, the order of the different systems presented in Fig. 4 and Table 2 is according to the order in which they appeared in the review.

**Table 2:** Conclusions and comparisons of the different PARRs (P: Pneumatic-driven; E: Electrically-driven)

Group/device	Institution/nationality	Actuator types	DOFs	ROM	Exercise mode	Control strategies	Subject	Study with patients
Girone et al. Rutgers Ankle	Rutgers University, USA	P	6	DO/PL:$\pm 45^{\circ }$ IN/EV:$\pm 40^{\circ }$ AD/AB:$\pm 80^{\circ }$	Passive; Active	Position control Force control	N = 4 [58]	yes
Yoon et al. Reconfigurable parallel ankle robot	Gwangju Institute of Science and Technology, Korea	P	4	DO/PL:$\pm 50^{\circ }$ IN/EV:$\pm 55^{\circ }$	Passive	Position control	-	no
Dai et al. “ $3{\mathrm{- }}SPS/PS$” mechanism	King’s College London, UK	E	4	–	–	–	–	no
Liu et al. “ $3{\mathrm{- }}RSS/S$” mechanism	Hebei University of Technology, China	E	3	DO/PL: ${\mathrm{- }}{41.86^{\circ }} \sim {42.79^{\circ } }$; IN/EV: ${\mathrm{- }}{43.8^{\circ }} \sim {41.89^{\circ } }$; AD/AB: ${\mathrm{- }}{53.78^{\circ }} \sim {44.08^{\circ } }$;	Passive; assistant; resistive	Position control Force control	–	No
Saglia et al. ARBOT	Istituto Italiano di Technologia, Italy	E	2	-	Passive Active Active (Isometric Isotonic)	Position control Assistive control Admittance control	N = 1 [27]; N = 5 [60]	No
Malosio et al. PKAnkle	Institute of Industrial Technologies and Automation, Italy	E	3	-	Passive Active	Position control Admittance control	N = 3 [86]	No
Ayas et al. 2-DOFs parallel ankle robot	Karadeniz Technical University, Turkey	E	2	-	Passive Active	Trajectory tracking Adaptive admittance control	–	No
Hamid et al. 9-DOFs hybrid PARR	University of Birmingham, UK	E	9	DO/PL: ${\mathrm{- }}{42.24^{\circ }} \sim {25.92^{\circ } }$; IN/EV: ${\mathrm{- }}{16.46^{\circ }} \sim {16.11^{\circ } }$; AD/AB: ${\mathrm{- }}{30.49^{\circ }} \sim {33.71^{\circ } }$;	Passive	Position control	–	No
Ai et al. 2-DOFs ankle rehabilitation robot	Wuhan University of Technology, China	P	2	-	Passive	Adaptive backstepping sliding mode control	N = 5 [52]	No
Jamwal et al. Reconfigurable PARR	University of Auckland, New Zealand	P	3	DO/PL:$\pm 46^{\circ }$ IN/EV:$\pm 26^{\circ }$ AD/AB:$\pm 52^{\circ }$	Passive	Trajectory tracking control	N = 1 [49]	No
Jamwal et al. intrinsically compliant ankle rehabilitation robot	Nazarbayev University, Kazakhstan	P	3	–	Passive Active	Position control Impedance control Adaptive impedance control	N = 10 [50]; N = 10 [68] N = 3 [69]	Yes
Zhang et al. CARR	University of Auckland, New Zealand	P	3	Varying workspace	Passive Active	Position control Adaptive patient-cooperative control Adaptive trajectory tracking	N = 1 [34] N = 4 [47] N = 4 [70] N = 2 [71]	Yes
Tsoi et al. Redundantly actuated PARR	University of Auckland, New Zealand	E	3	DO/PL: ${\mathrm{- }}{60^{\circ }} \sim {72^{\circ } }$; IN/EV:$\pm 73^{\circ }$; AD/AB:$> 80^{\circ }$;	Passive Active	Joint force control Impedance control	N = 1 [64]	No
Wang et al. $3-\underline{R}US/\underline{R}RR$	Beijing Jiaotong University, China	E	3	DO/PL: ${\mathrm{- }}{62^{\circ }} \sim {50^{\circ } }$; IN/EV:$\pm 37^{\circ }$; AD/AB:$\pm 92^{\circ }$;	Passive	Position control	-	No
Cazalilla et al. $3{\mathrm{- }}\underline{P}RS$	Universidad Politécnica de Valencia, Spain	E	3	DO/PL:$\pm 50^{\circ }$; IN/EV:$\pm 50^{\circ }$;	Passive Active (assistive; resistive)	Position control Force control	–	No
Li et al. 2-UPS/RRR PARR	Beijing University of Technology, China	E	3	DO/PL: ${\mathrm{- }}{42.24^{\circ }} \sim {25.92^{\circ } }$; IN/EV: ${\mathrm{- }}{16.46^{\circ }} \sim {16.11^{\circ } }$; AD/AB: ${\mathrm{- }}{30.49^{\circ }} \sim {33.71^{\circ } }$;	Passive; Active	Position control Patient-passive compliance exercise Isotonic exercise Patient-active exercise	N = 5 [67]	No

### Future solutions to improve the PARRs

Ankle rehabilitation training is a small branch of rehabilitation robotics. Very significant results have been achieved in upper-limb rehabilitation robots [76] and lower-limb rehabilitation robots [77, 78]. The state-of-the-art related technologies for rehabilitation robots can provide references for the development of PARRs. According to the research status of ankle rehabilitation robots and research progress in the field of rehabilitation robots, in our opinion, future research on PARRs will focus on mechanism optimization, compliance control with variable parameters, multimode rehabilitation training methods, multimodal motion intention recognition, and the evaluation and selection of the optimal exercise therapy.

#### Mechanism optimization

Existing PARRs all have their own disadvantages, such as insufficient or redundant DOFs, the rotation center of the AJC not coinciding with the mechanism’s rotation center while ankle rehabilitation requires varying positions of the AJC and synergistic movement of the lower limb from the patient, a complex mechanism with branches that easily lead to interference, poor driving modes and inconvenient use. Therefore, designing a PARR with only three DOFs and whereby the mechanism’s rotation center coincides with the AJC rotation center, which also has a simple structure, enough workspace, ease of use and small size, is a key problem in the mechanism design and optimization of PARRs. In addition, the actuator types are very important. As we discussed, the pneumatic-driven PARRs have the advantage of intrinsic compliance, but they are difficult to control precisely owing to the nonlinear and time-varying characteristics of PMAs, while the electrically-driven PARRs can be controlled precisely but lack the intrinsic compliance and require complex compliance control algorithms. Therefore, the design of actuators combining the advantages of the two actuator types will be a research hotspot, and some new actuators may be used for the development of PARRs.

#### Compliance control with variable parameters

Many PARRs have been developed with compliance control by using impedance control, admittance control and their derivative compliance control algorithms. However, it is not convenient to use compliance control strategies with fixed parameters because the mechanical impedance of the AJC changes both with the stage of rehabilitation training and significantly between patients. Although some researchers have studied adaptive admittance control [67, 71] and adaptive impedance control [69], a method combining the impedance/admittance control strategies and techniques with rehabilitation robot prototypes in 3-DOFs is still lacking. In particular, the compliance control strategies with variable impedance parameters or admittance parameters are still in the stage of simulation and laboratory experiments. Compliance control with variable parameters should be further studied, and the ability to adaptively adjust parameters based on the degree of AJC dysfunction, the degree of regained AJC ROM and the degree of regained ankle muscle strength will be of vital importance. Specifically, the existing compliance algorithms with variable parameters are complex in calculation and have poor real-time performance [69, 71], as the adaptation laws are based only on the measured human-robot interaction force/torque. The surface electromyography (sEMG) signal utilized to adjust the impedance or admittance parameters will be a research trend for the sEMG signals have strong real-time performance and can be complementary with force/torque sensors.

#### Multimode rehabilitation training methods

The current rehabilitation training methods of PARRs are almost all within the control framework shown in Table 1 [28, 60] and mainly involve passive rehabilitation training based on position control and different active rehabilitation trainings based on position control and impedance/admittance control such as assistive, resistive, isotonic and isometric exercises. Passive rehabilitation training combined with active rehabilitation training can help patients regain their AJC ROM and muscle strength. However, there are still some important rehabilitation methods that have not been studied, such as isokinetic exercise, eccentric exercise, concentric exercise and proprioceptive exercise, which have been developed in upper-limb rehabilitation robots and lower-limb rehabilitation robots. In particular, there is no real-time online detection system to judge the degree of ankle rehabilitation and to determine the optimal rehabilitation training methods at different stages in real time; this system could better help restore the functions of the AJC.

#### Multimodal motion intention recognition

From the above review, we can see that none of the PARRs use sEMG signal information for active rehabilitation. A commonly used sensor in PARRs at the present stage is the force/torque sensor [60, 67, 71]. However, visible movement of the limb always occurs after the human body intends to move. The prediction result is simply the byproduct of the behavioral intention, not the actual behavioral intention, whereas sEMG signals can truly reflect the movement intentions of the human body because muscle and nerve signals are produced prior to force generation, known as electromechanical delay [77]. There have been many studies involving sEMG for motion intention recognition in the application of rehabilitation robots [79–81], and the sEMG signal should be incorporated in the active exercise of the AJC to make the active exercise more real-time and comfortable even though the sEMG signals change due to a variety of factors, such as placement, fatigue and sweat.

#### Evaluation and selection of the optimal exercise therapy

PARRs are used to help patients regain their limited ROM, restrength their weak muscles, recover their dynamic balance, and restore their motion functions. However, most PARRs have been tested using only healthy subjects, while few have been tested on patients with AJC dysfunctions [69–71]. Therefore, the current research deviates from clinical application. In addition, therapy should be tailored to each patient’s needs and abilities to avoid “one-size-fits-all” treatment because the ROM and muscle strength are different for different patients. Therefore, evaluating the degree of dysfunction and developing optimal exercise therapy sessions according to the patient and the recovery stage are of vital importance and have not been researched in combination with clinical trials. This will be a research trend if a PARR is developed for clinical rehabilitation and not just scientific research.

### Limitations of this review

This study aims to provide a systematic review of the state of the art in PARR technology. Therefore, ankle rehabilitation robots without parallel mechanisms, such as the ankle-foot orthosis (AFO) [82, 83, 87, 88], the 1-DOF robotic ankle rehabilitation system [84, 85, 89], and those not for ankle rehabilitation [90] are excluded. Only articles published after 1999 were included in this study, and the literature search was performed using PubMed, Scopus, IEEE Xplore, and Web of Science. Therefore, other studies before 1999 or in other databases may exist that were not found. In addition, only papers written in English and accessible to the authors were included in the study. Some studies may have not been included on the basis of this search strategy, resulting in a potentially incomplete search.

## Conclusions

This review mainly summarized the state-of-the-art PARRs in terms of pneumatic-driven and electrically driven PARRs, with consideration of the mechanism configurations, actuator types with different trajectory tracking control techniques and rehabilitation training methods. By comparing, analyzing and summarizing the differences and similarities among the reviewed PARRs, we can see that more work needs to be done to perfect the mechanical design and interactive control strategies to maximize patient safety and improve rehabilitation outcomes, although significant progress has been made in the development and improvement of PARRs over the last two decades, with the mechanical designs and control strategies being greatly improved for both pneumatic-driven and electrically driven PARRs. Designing a PARR with only three DOFs and whereby the mechanism’s rotation center coincides with the AJC rotation center is of vital importance in the mechanism design and optimization of PARRs. For the control strategy, compliance control with variable parameters should be further studied, with sEMG signal included to improve the real-time performance. And multimode rehabilitation training methods with multimodal motion intention recognition, real-time online detection and evaluation system should be further developed to meet the needs of different ankle disability and rehabilitation stages. In the foreseeable future, there will be PARRs that combine the best of both pneumatic-driven and electrically driven PARRs. The research hotspots and trends in this field will mainly center on mechanism optimization, compliance control with variable parameters, multimode rehabilitation training methods specific to different ankle disabilities and rehabilitation stages, multimodal motion intention recognition, and the evaluation and selection of the optimal exercise therapy.

The other thing to note is that few of the developed PARRs have been subjected to clinical trials, all the control strategies and rehabilitation training modes are in the stage of testing, with none being used in clinical applications. Some simple ankle rehabilitation devices have already been in commercial use, such as the AnkleMotus from Shanghai Fourier Intelligent Technology Co., Ltd, China, and the Minitalus from EasyTech, Italy. However, they were designed for training with a specific function or for a single AJC’s DOF. The PARRs with more comprehensive rehabilitation training function are developing rapidly, and several are already in clinical trials. It is expected that within a year or two, PARRs with full rehabilitation training capabilities will be implementable as an intervention in clinical practice.

Generally speaking, the study provides a systematic review of the PARRs’ researches and technologies. Our goal in this review is to guide future researchers in the development of better devices for patients with ankle dysfunctions. We hope that this review will serve as a useful resource for future developers and facilitate the evolution of the field.

## Data Availability

All data generated or analyzed during this study are included in this published article.

## Abbreviations

AJC: Ankle joint complex
CNS: Central nervous system
PARR: Parallel ankle rehabilitation robot
ROM: Range of motion
DOF: Degree of freedom
DO/PL: Dorsiflexion/plantarflexion
IN/EV: Inversion/eversion
AD/AB: Adduction/abduction
T: Translation
R: Revolute joint
S: Spherical joint
P: Prismatic joint
U: Universal joint
IIT: Istituto Italiano di Tecnologia
SPR: Soft parallel robot
PMA: Pneumatic muscle actuator
CARR: Compliant ankle rehabilitation robot
FIS: Fuzzy inference system
PMS: Physiological motion space
FBDO: Fuzzy-based disturbance observer
NIFT: Normalized iterative feedback tuning
ABS-SMC: Adaptive backstepping sliding mode control
RMSD: Root mean square deviation
NRMSD: Normalized root mean square deviation
FOPID: Fractional order PID
PID: Proportion integration differentiation
EMG: Electromyographic
sEMG: Surface electromyography
MIMO-DCSMC: Multi-input–multi-output disturbance compensated sliding mode controller
BFSGA: Biased Fuzzy Sorting Genetic Algorithm
DE: Differential evolution
